# Effect of Cognitive Function on Balance and Posture Control after Stroke

**DOI:** 10.1155/2021/6636999

**Published:** 2021-01-28

**Authors:** Hui-xian Yu, Zhao-xia Wang, Chang-bin Liu, Pei Dai, Yue Lan, Guang-qing Xu

**Affiliations:** ^1^Department of Rehabilitation Medicine, Beijing Tiantan Hospital, Capital Medical University, Beijing 100060, China; ^2^China National Clinical Research Center for Neurological Diseases, Beijing 100060, China; ^3^Department of Rehabilitation Medicine, Guangzhou First People's Hospital, School of Medicine, South China University of Technology, Guangzhou 510050, China; ^4^Department of Rehabilitation Medicine, Guangdong General Hospital, Guangdong Academy of Medical Sciences, 510080, China

## Abstract

Hemiplegic gait is the most common sequela of stroke. Patients with hemiplegic gait are at a risk of falling because of poor balance. The theory of cognitive-motor networks paved the way for a new field of research. However, the mechanism of the relationship of cognition with gait or posture control networks is unclear because of the dynamic characteristics of walking and changing postures. To explore differences in the balance function and fall risk between patients with and without cognitive impairment after stroke, we utilized the Berg balance scale, Timed “Up and Go” test, and 10 m walking test. Patients were divided into two groups: the observation group (16 patients, female 6 and male 10), comprising patients with cognitive impairment after stroke, and the control group (16 patients, female 7 and male 9), comprising patients without cognitive impairment after stroke. We found that patients with cognitive impairment had worse balance function and a higher risk of falls. They needed a longer time to turn around or sit down. Our findings indicated that posture control in turning around and sitting down required more cognitive resources in daily life.

## 1. Introduction

Approximately 60% to 80% of patients after stroke cannot ambulate independently after completion of rehabilitation, and many of them have hemiplegic gait, which limits motor function [1]. Decreased balance and gait after stroke are the most common factors of decreased activities of daily living (ADL). Falls are a major adverse event in daily care. Approximately 60% of patients fall at least once during hospitalization, while 73% of them fall in the first 6 months after stroke [2]. Falling can increase the patient's fear of falling, restrict activity, and increase dependency [3]. A fall can enhance social dependence, cause serious anxiety and depression [4], and prolong hospitalization. It can also cause hip fractures, necessitating long-term wheelchair use after stroke [5].

In earlier reports, most researchers thought that our actions were controlled by the motor system alone. However, recently, many researchers have found that a sensory-cognitive-motor network system is necessary to ascertain the accuracy of an action [6–8]. The sensory-cognitive-motor neural circuits involve the frontal cortex, subcortex, basal ganglia (caudate nucleus, globus pallidus, and thalamus), brainstem, and cerebellum [9]. They can ensure safe and effective posture changing for adaptation to complex environments at home or in a community. Cognition is associated with gait velocity [10]. Evidence has been increasing on the associations between cognitive function and balance in the elderly patients with Alzheimer's or Parkinson's disease [11, 12]. However, limited research has focused on this relationship after stroke. Elucidating the relationship between the brain areas for motor function and cognition is important for establishing rehabilitation strategies. Our assumptions on balance and posture control guide how we assess and treat balance and posture control disorders. Therefore, understanding the mechanisms of cognitive function in posture control and coordination could help us improve balance and reduce the incidence of falls. This study was aimed at observing the effects of cognitive function on balance and fall during complex motor skills for ADL performed by patients after stroke.

## 2. Methods

### 2.1. Patients

A retrospective case-control study design was used. We collected the data of 32 patients with hemiplegic gait after stroke. Patients were divided into two groups: the observation group, comprising patients with cognitive impairment (CI), and the control group, comprising patients without CI. From the department of rehabilitation from May 2019 to January 2020, we randomly selected 16 patients, including ten men and six women, with a Montreal Cognitive Assessment (MoCA) score of 15–25, and 16 patients, including nine men and seven women, with an MoCA score ≥ 26. Inclusion criteria were (1) the definitive diagnosis of first-ever stroke, (2) localization of stroke in the basal ganglia region of the right or left hemisphere, (3) age of 40–60 years, (4) ≤3 months poststroke, (5) junior high school education, (6) lower-limb Fugl-Meyer motor assessment score of 20–30, and (7) independent walking for several meters. Exclusion criteria were (1) prestroke vascular dementia, (2) aphasia, (3) traumatic hemorrhage, (4) sensory impairment, (5) medical instability hindering participation, and (6) visual or hearing impairment.

### 2.2. Screening Measures

MoCA was first developed by Nasreddine et al. [13]. It is a brief screening tool to evaluate cognitive and attentive/executive functions and has also been used in studies on executive function assessment [14]. The scale includes eight cognitive domains with a best potential score of 30 points. Each correct answer accounts for 1 point, while a wrong answer or no answer accounts for 0 point.

### 2.3. Outcome Measurement

The Berg balance scale (BBS) was used to assess balance function for activity limitations [15]. It is a consensus measurement of the International Classification Functioning (ICF) activity on a five-point scale, ranging from 0 to 4, where “0” indicates the lowest level of function and “4” the highest, with a total score of 56.

**Figure 1:**
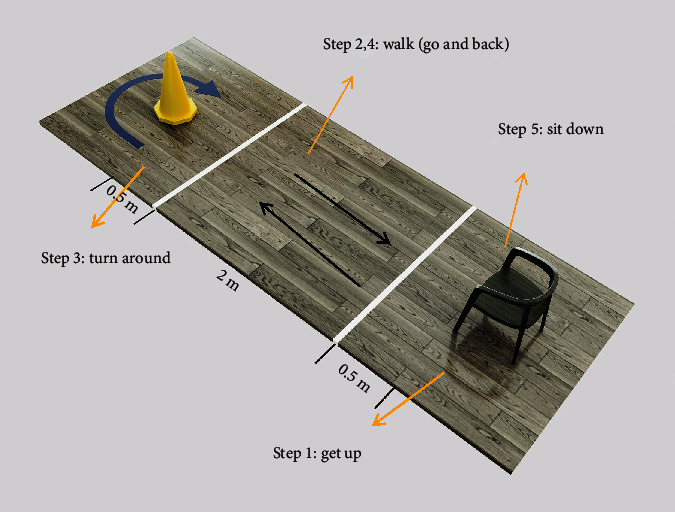
Timed “Up and Go” test was divided into four periods based on the posture: steps 1, 2+4, 3, and 5. The times in the four periods are shown.

The fall risk was assessed with the Timed “Up and Go” test (TUGT) [16], constituting five steps: getting up, walking, turning around, walking, and sitting down (Figure 1). Patients were asked to stand up from a standardized armchair, walk 3 m (marked by two tapes: one, 0.5 m from the chair; another, 0.5 m from the cone), turn around the cone, and sit down on the chair safely. To clarify the difference in each posture condition between the two groups, we divided the process into four periods: getting up, walking straight, turning around, and sitting down. Before testing, participants sat in the chair. The tester recorded the time taken to complete the four processes with a stopwatch. Getting-up time (GT) was the time between the patient's back leaving the chair and the patient reaching the line drawn 0.5 m from the chair. Walking-straight time (WT) was the time between the patient reaching the line 0.5 m from the chair and the patient reaching the line 0.5 m from the cone. Turning-around time (TT) was the time between the patient reaching the line 0.5 m from the cone and the patient finishing the turning and crossing the line 0.5 m from the cone. Sitting time (ST) was the time between the patient reaching 0.5 m from the chair and the patient sitting down again with their back to the chair. The total time of “get up and go” is TGUG.

The 10 m walking test (10MWT) was used to assess the walking function. Patients walked from the taped line on the floor to an invisible line drawn 10 m away, which they were unaware of. Testers recorded the time with a stopwatch between the patient's first step crossing the line and the patient's first leg crossing the invisible line [17].

### 2.4. Data Analysis

The statistical analysis and graphing were performed using GraphPad Prism 8.0 (GraphPad Software, Inc., San Diego, California, USA). Continuous variables are expressed as the mean ± standard deviation. The unpaired *t*-test was performed to evaluate between- and within-group differences before and after the treatment of patients in the observation and control groups. A *P* value < 0.05 was considered statistically significant.

## 3. Results

**Figure 2:**
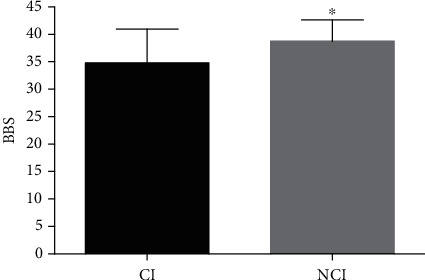
Differences in Berg balance scale scores between the observation and control groups. A *P* value < 0.05 was considered to indicate statistical significance.

**Figure 3:**
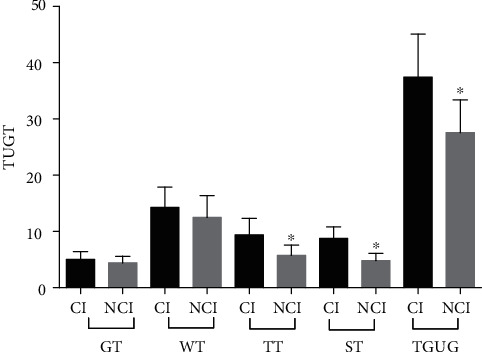
Differences in the overall time in the Timed “Up and Go” test and the times of the four periods of getting up, walking straight, turning around, and sitting down between the observation and control groups. ^∗^A *P* value < 0.05 was considered to indicate statistical significance. GT: getting-up time; WT: walking-straight time; TT: turning-around time; ST: sitting time; TGUG: the total time of “get up and go.”

**Figure 4:**
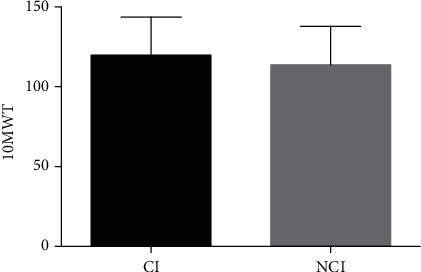
Differences in 10 m walking test between the observation and control groups. A *P* value > 0.05 was considered to not indicate statistical significance.

Age, sex, onset time, lesions, or scores of the lower-limb Fugl-Meyer motor assessment did not differ from baseline in either group, and there was no difference in baseline data between the groups (Table 1). BBS scores were significantly lower in the observation group than in the control group (*P* = 0.039; Figure 2). As for TUGT, the overall time was significantly longer in the observation group than in the control group (*P* = 0.005; Figure 3). GT or WT did not significantly differ between the groups (GT, *P* = 0.18; WT, *P* = 0.19). TGUG, TT, and ST were significantly longer in the observation group than in the control group (TGUG, *P* = 0.005; TT, *P* = 0.003; and ST, *P* = 0.002). The 10MWT scores did not differ between the two groups (*P* = 0.48; Figure 4).

## 4. Discussion

This study showed that patients with poor cognitive function had worse balance and posture control. Compared to walking straight, turning around and sitting down required more cognitive resources. Patients with hemiplegia with CI had a greater risk of fall during turning around or sitting down. These results suggested that we should pay more attention to the training of turning around or sitting down in the balance training of patients with CI after stroke.

The BBS is commonly used to evaluate balance function after stroke. Patients with CI had significantly poorer balance function than those without CI. This may be associated with decreased executive function, which is subsumed by frontal regions and is the most common type of impairment in cognitive function. Pahlman et al. reported that differences in BBS scores between patients with and without executive dysfunction were significant in the first year poststroke [18].

Although some studies reported that cognition has a mediating effect on some associations between gait velocity and volumes [6, 19, 20], the present study showed no significant difference in 10MWT results between the two groups. It is currently unclear if cognition directly affects gait velocity and if impairments in both gait and cognition result from changes within the brain. Our understanding of the interactions among gait, cognition, and brain, and whether or not it applies to gait characteristics other than velocity, is limited because of the scarcity of studies assessing cognition in addition to gait with neuroimaging parameters [21]. Walking in the community can be more challenging for stroke survivors. Our results suggested that declined cognitive function would increase the risk of falling during turning around and sitting down after stroke. It is unclear if there is a direct relationship between gait and cognition, if CI directly impacts gait velocity, or if CI and hemiplegic gait are concurrent sequelae due to damaged cognitive-gait neural circuits after stroke. Our understanding is limited on the interactions among gait, cognition, and cognitive-gait neural circuits and on which gait characteristics would manifest with CI after stroke.

Unlike walking in a straight line, the control of balance during gait and changing postures (getting up, walking, stopping, turning around, and sitting down) requires a complex control of the center of body weight. The TUGT, which detects the risk of falls in individuals with stroke, includes several parts of autonomous posture control, such as the movement from sitting to standing (which requires the participation of expected posture adjustment ability), continuous walking (which requires dynamic control ability), and turning and sitting (which requires participation of spatial orientation ability) [22]. The present study showed significant differences in TUGT scores between the observation and control groups. The overall time was significantly longer in the observation group than in the control group. In TUGT, because different positions were timed separately, we could observe the time difference between the two groups in different postures. Further, ST and TT significantly differed between the two groups. The results indicated that sitting (walking to the chair, turning around, determining the height of the chair, and sitting down smoothly) and turning (stopping the walk, noticing the cone, and steering around the obstacle safely) required greater spatial orientation and attention ability based on the interpretation of convergent sensory information from the somatosensory, vestibular, and visual systems. There was no difference in WT and GT between the two groups. The results indicated that walking in a straight line and getting up required less cognitive resources.

Falls after stroke are common [23]. There are many factors that lead to fall in stroke patients, including impaired balance and damaged posture control. Postural control refers to the control of the body's position in space for the purpose of stability and orientation. Postural orientation is the ability to maintain appropriate relationships between the different body segments and the task environment, with the help of the vestibular system, the somatosensory system, and the visual system. Postural stability, also known as balance, is the ability to control the relationship between the center of mass and the base of support. Posture to adapt to the environment requires the perception, spatial orientation, and the ability to pay attention to environmental changes. Postural orientation determines the position of objects, actively aligns the trunk and head with respect to gravity, and considers surfaces, visual surroundings, and internal references. In ADL or community activity, patients need complex posture control abilities (including posture stability and orientation) for adaptation to the complex environments to avoid falling. A safe, smooth, and coordinated motion is regulated by the sensory-cognitive-motor system. The viewpoint is inaccurate that the motor system is sufficient in controlling effective motions. Every motion involves a highly complex skill-posture control in the process of cognition for adaption to the current environment [24]. While in walking, people generally prepare for the walk, stop, change the speed, turn, perform tasks, sit down, and so on. The two main functional goals of postural behavior are orientation and equilibrium.

An inappropriate preparation can cause a fall. However, studies are limited on accurate postural assessment, balance assessment, or resource allocation of cognitive function after stroke. Mechanisms mediating the preparation of coordinated motor function have not been clearly elucidated. It is important to understand the potential preparatory mechanisms for coordinated actions. Motor mechanisms in the brain are viewed as a slave system under the dictate of cognition. Accordingly, modality-specific sensory modules channel information to the central systems for attention, memory, language, concepts, and decisions, which, in turn, drive the motor output [25]. Previous studies have suggested that hemiplegic gait and poor balance function lead to falls. Recently, increasing evidence has suggested that cognitive function is involved in complex motor and posture control and that posture control during walking receives instructions from the cognitive brain regions. The prefrontal cortex is the central brain region for cognitive function. It is strongly associated with the primary motor cortex, motor area cortex (M1 area), subcortical structures, and cerebellum [26]. The regions, whether anatomical connections or functional network connections, related to gait posture control are uncertain. Some studies have focused on one or several regions, but a very few studies have assessed regions connected through structural tracts, which connect brain regions of posture control and cognition [18]. A study assessed the functional networks connecting the brain regions of cognition and gait posture control [27]. The basal ganglia region is the most common site of stroke. Studies using neuroanatomy, neurophysiology, and functional magnetic resonance imaging have demonstrated that the cortical motor areas, basal ganglia, and cerebellum not only are associated with motor control but also play roles in cognition, language processing, perception, and learning [28]. The dorsolateral prefrontal cortex (the anatomical origin of cognitive function) connects with the striatum, basal ganglia, and thalamus through white matter tracts [29]. This frontal-subcortical circuit links cognitive and motor brain processes to adapt with environment. These impairments impact an individual's abilities to perform ADL and are associated with an increased risk of falls.

The simple view of a balance system that one or a few balance centers in the central nervous system were responsible to control balance is limiting and can partially account for our limited abilities to assess risks of falling accurately, to improve balance, or to reduce falls. Cognitive dysfunction, particularly executive dysfunction, is obscured by other sequelae after stroke. Mild CI may often be overlooked during evaluation in clinical settings [30]. With further studies on cognitive-motor functional brain networks, the new theory of balance and posture control could improve, and rehabilitation strategies could be more accurate. In our present study, based on the new cognitive-motor brain function network theory, we observed the important effect of cognitive function on postural control. Cognitive training may be a new therapeutic target to improve postural control after stroke.

Our study had some limitations. First, it was a retrospective study, which would provide a lower level of evidence than a randomized controlled trial. Second, we grouped patients only based on MoCA as the screening tool. In our subsequent studies, different cognitive domains were evaluated to understand the influence of different cognitive disorders on postural control. Finally, we did not collect the executive function, spatial orientation, and attention separately to clarify the effects of different cognitive functions on posture control and gait. We will continue to explore the characteristics of postural control disorders with CI after stroke. At the same time, in our further research (already ongoing), functional magnetic resonance imaging will be used to scan the brain of patients with or without CI after stoke. We will analyze the data, observe the activation of the regions of interest, and finally explore the cognitive-postural control brain network to find new therapeutic targets.

## 5. Conclusions

Patients with poststroke CI had poorer balance and a higher risk of falls. In daily activities, the risk of falls is significant during posture control, including turning and sitting. Our study suggested that posture control in turning around and sitting down required more cognitive resources in daily life. In physical therapy of balance and motor control after stroke, the assessment of cognitive function should not be neglected.

## Figures and Tables

**Table 1:** The baseline data.

Group	Age (years)	Sex (number)	Hemiplegic limb (number)	Onset time (month)	Fugl-Meyer (lower-limb)
Control group	52.31 ± 8.56	Female 7 Male 9	Left 8 Right 8	2.33 ± 0.24	23.21 ± 7.34
Observation group	51.56 ± 11.03	Female 6 Male 10	Left 9 Right 7	3.12 ± 0.98	20.97 ± 9.56

## Data Availability

If you need our data, please contact the corresponding authors.
